# Impact of type of reconstructed residence on social participation and mental health of population displaced by disasters

**DOI:** 10.1038/s41598-021-00913-3

**Published:** 2021-11-02

**Authors:** Tomomi Suzuki, Tetsuya Akaishi, Harumi Nemoto, Yusuke Utsumi, Moe Seto, Hitomi Usukura, Yasuto Kunii, Yumi Sugawara, Naoki Nakaya, Tomohiro Nakamura, Naho Tsuchiya, Akira Narita, Mana Kogure, Atsushi Hozawa, Ichiro Tsuji, Tadashi Ishii, Hiroaki Tomita

**Affiliations:** 1 Department of Disaster Psychiatry, Tohoku University Graduate School of Medicine, Sendai, Japan; 2 Health Promotion Section, Shichigahama Town Hall, Shichigahama, Japan; 3 Graduate School of Comprehensive Human Sciences, University of Tsukuba, Ibaraki, Japan; 4 Department of Education and Support for Regional Medicine, Tohoku University Hospital, Seiryo-machi 1-1, Aoba-ku, Sendai, Miyagi 980-8574 Japan; 5 Department of Psychiatry, Tohoku University Graduate School of Medicine, Sendai, Japan; 6 Department of Psychiatry, Tohoku University Hospital, Sendai, Japan; 7 Department of Disaster Psychiatry, International Research Institute of Disaster Science, Tohoku University, Sendai, Japan; 8 Department of Epidemiology, Tohoku University Graduate School of Medicine, Sendai, Japan; 9 Department of Preventive Medicine and Epidemiology, Tohoku Medical Megabank Organization, Tohoku University, Sendai, Japan

**Keywords:** Epidemiology, Health policy

## Abstract

After disasters, people are often forced to reconstruct or move to new residences. This study aimed to reveal the association between the types of reconstructed residences and psychosocial or psychiatric conditions among the population. A total of 1071 adult residents in a coastal town, whose houses were destroyed by the tsunami caused by the Great East Japan Earthquake, enrolled in the study five years after the disaster. The type of reconstructed post-disaster residences (reconstructed on the same site/disaster-recovery public condominium/mass-translocation to higher ground/privately moving to remote areas) and the current psychosocial indicators were investigated. The results revealed that individuals living in public condominiums showed significantly worse scores on the Lubben Social Network Scale-6 (p < 0.0001) and the Center for Epidemiologic Studies Depression Scale (p < 0.0001), and slightly worse scores on the Kessler Psychological Distress Scale (p = 0.035) and the Impact of Event Scale-Revised (p = 0.028). Lower psychosocial indicator scores in the public condominium group were more remarkable in younger adults aged < 65 years. Insomnia evaluated using the Athens Insomnia Scale was not different among the four residential types. In summary, residents moving into disaster-recovery public condominiums are likely to have less social interaction, be more depressed, and may need additional interventions.

## Introduction

Interpersonal relationships and active communication are closely related to physical and mental status in a local community^1,2^. Both for the general population, including children and older adults, and people suffering from physical disorders or neuropsychiatric disorders such as depression and dementia, social isolation negatively affects physical and mental health^2,3^. Active social involvement and a positive attitude toward social networks have been reported to be important for maintaining the mental health status and well-being of disaster-affected populations or patients with physical disabilities^4,5^. Furthermore, an active social lifestyle and interpersonal communication in social networks are reported to decrease the risk of dementia and depression^6,7^. It is expected that a strong social support and relationships will prolong a healthy life expectancy^8,9^.

After large-scale disasters, such as the Great East Japan Earthquake (GEJE) in 2011, many people are dislocated from their homes, and personal relationships in the local community are largely disturbed. Many reports have shown that long-term displacement of disaster-affected people may negatively influence their psychosocial and psychiatric status^10–12^. Although the forced evacuation after large-scale disasters has been established as increasing the risk to physical and mental well-being, the potential impact of the types of reconstructed residences on the long-term psychosocial and psychiatric status of disaster-affected populations has not been fully elucidated.

Shichigahama Town is a coastal town and one of the towns closest to the epicenter of the earthquake. Since the occurrence of the earthquake, Tohoku University and Shichigahama Town have officially entered into a contract for promoting health in the town, which was named the Shichigahama Health Promotion Project^12,13^. The town was inhabited by approximately 20,000 residents before the earthquake. About one-fourth of the town’s area was hit by the tsunami and submerged in water. The houses of more than 2000 residents were destroyed by the tsunami. Several days after the earthquake, the number of evacuees in the town peaked at more than 6000 people. Many of the evacuees were forced to stay in refugee camps, which were established in public community centers or stadiums of the schools in town, for more than several weeks to months. In the following years, most of the dislocated population reconstructed their houses or moved into the officially built disaster-recovery public condominiums. This cross-sectional study was conducted in Shichigahama Town in November 2016 (i.e., five years after the GEJE). The residents whose houses were destroyed by the tsunami were enrolled, and data regarding the types of reconstructed residences, their current social interaction activities, and mental health conditions were evaluated.

This study aimed to elucidate the relationship between the types of reconstructed residences and the subsequent psychosocial and psychiatric conditions among disaster victims. By elucidating the relationship, this study further aimed to support the local governments’ policy instituted after large-scale disasters regarding the reconstruction of disaster-relief residences for the affected community members. To estimate the current psychosocial and psychiatric conditions among disaster-affected population, the scores of the following psychosocial indicators were comprehensively collected: the Lubben Social Network Scale (LSNS-6), the six-item version of the Kessler psychological distress scale (K6), the Center for Epidemiologic Studies Depression Scale (CES-D), the Impact of Event Scale-Revised (IES-R), and the Athens Insomnia Scale (AIS)^14–18^. These scores among the victims were then evaluated according to the types of reconstructed residences, together with other several socio-economic or disaster-exposure factors that may potentially affect the scores of the indicators.

## Results

### Participants

**Figure 1:**
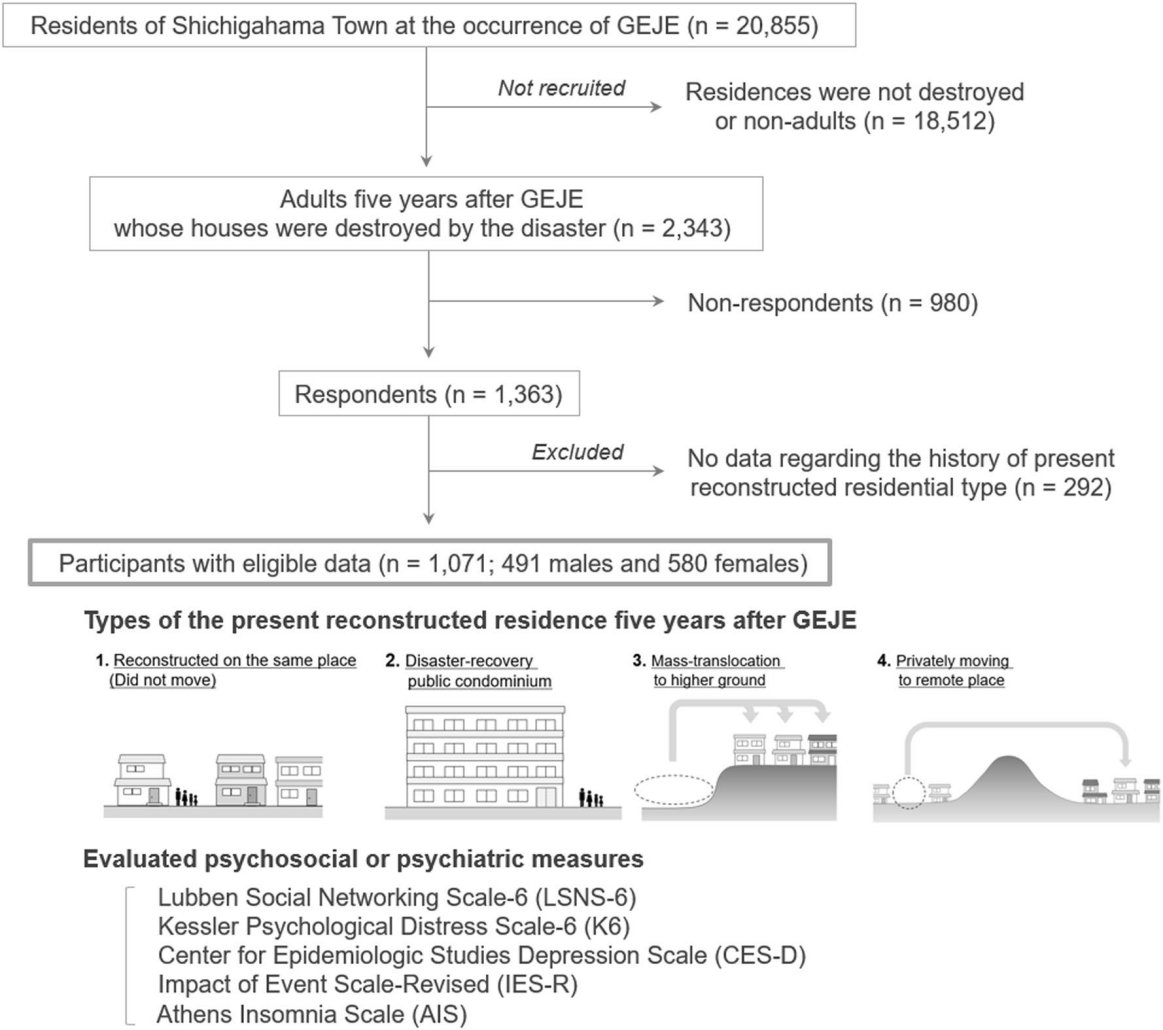
Flow diagram of the study design. Shichigahama Town was one of the closest coastal cities or towns to the epicenter, where more than 10% of the residents lost their homes because of the tsunami. All 2343 residents whose homes were destroyed were initially recruited, of whom 58.2% responded and participated in the present study. Illustrations of the four different types of reconstructed residences five years after the earthquake and tsunami are shown, which were classified into: (1) those which did not move (reconstructed on the same site), (2) disaster-recovery public condominium, (3) mass-translocation to higher ground, and (4) private movement to remote areas. *GEJE* 2011 Great East Japan Earthquake.

Adult residents (aged ≥ 18 years in 2016) of Shichigahama Town (n = 2343), whose houses were destroyed by the tsunami, were initially recruited for this study. Among them, 1363 residents (response rate: 58.2%) provided written informed consent. From these, 292 people were excluded because of missing answers regarding their current type of reconstructed residence. The data of the remaining 1071 residents who answered the question regarding their current type of residence were considered eligible for this study. The flow diagram of the study design and the number of participants in each step are shown in Fig. 1. The eligible adults were asked to choose their current type of residential reconstruction five years after the disaster from among the following four categories: (type 1) reconstructed residence where they used to live at the occurrence of the disaster (i.e., did not move), (type 2) moved into a disaster-recovery public condominium, (type 3) massively relocated to higher grounds, and (type 4) privately moved to another area, remote from where they used to live.

### Types of the current reconstructed residence

The demographic features and outcomes evaluated by the types of current residences are listed in Table 1. From the eligible 1071 participants, 361 (33.7%) reconstructed their houses on the same site as before the GEJE (i.e., did not move), 118 (11.0%) moved into a disaster-recovery public condominium, 289 (27.0%) joined mass-translocation to higher grounds with neighbors, and 303 (28.3%) privately moved to remote areas. The ratio of sex and age did not differ between the four types of reconstructed residences. The number of family members per household was significantly lower in people who moved into disaster-recovery public condominiums (p < 0.01, Kruskal–Wallis test).

**Table 1:** Demographic data, social interaction, and mental health conditions by the current residential types 5 years after the GEJE.

	Type of the current reconstructed residence				p-value
	Did not move	Public condominium	Mass-translocation to higher ground	Privately moving to remote areas	
Number of participants, n	361	118	289	303	-
Male/Female, n	177 / 184	50 / 68	126 / 163	138 / 165	0.4493
Age* (years)	54.4 ± 17.9 years	56.3 ± 17.8 years	52.5 ± 18.3 years	54.1 ± 17.7 years	0.2511
Number of people per household^†^, n	3 (2–5)	2 (2–3)^‡^	4 (3–6)	4 (3–5)	< 0.0001
Current employment, n (%)	216/361 (59.8%)	51/118 (43.2%)	167/289 (57.8%)	163/303 (53.8%)	0.0123
Decreased income after GEJE, n (%)	77/226 (34.1%)	26/55 (47.3%)	58/175 (33.1%)	63/166 (38.0%)	0.2316
History of living in prefabricated temporary housing, n (%)	119/282 (42.2%)	97/106 (91.5%)	208/262 (79.4%)	158/261 (60.5%)	< 0.0001
**Scores and abnormal rates of mental health-related self-reported questionnaires**					
LSNS-6*	15.2 ± 5.8	12.7 ± 5.4^‡^	15.7 ± 5.6	15.1 ± 5.6	< 0.0001
LSNS-6 score < 12, n (%)	90/356 (25.3%)	47/112 (42.0%)	65/283 (23.0%)	75/297 (25.3%)	0.0011
K6^†^	2 (0–6)	4 (0–7)	2 (0–6)	2 (0–6)	0.0345
K6 score ≥ 13, n (%)	8/345 (2.3%)	8/114 (7.2%)	12/280 (4.3%)	13/293 (4.4%)	0.1398
CES-D*	13.0 ± 7.3	17.0 ± 9.1^‡^	12.7 ± 8.2	12.5 ± 8.2	< 0.0001
CES-D score ≥ 16, n (%)	78/272 (28.7%)	36/86 (41.9%)	58/217 (26.7%)	61/224 (27.2%)	0.0510
IES-R^†^	10 (2–21.5)	12.5 (4–28)	8 (2–21)	8 (2–18)	0.0281
(Intrusion)	3 (1–8)	4 (1–10)	3 (0–7)	2 (0–7)	0.0225
(Avoidance)	4 (0–8)	4 (1–10)	3 (0–8)	3 (0–8)	0.0249
(Hyperarousal)	3 (0–6)	4 (1–7)^‡^	2 (0–6)	2 (0–5)	0.0150
IES-R score ≥ 25, n (%)	58/323 (18.0%)	30/110 (27.3%)	45/260 (17.3%)	44/278 (15.8%)	0.0636
AIS*	4.1 ± 3.6	4.3 ± 4.0	3.9 ± 3.5	3.9 ± 3.4	0.7572
AIS score ≥ 6, n (%)	101/353 (28.6%)	35/110 (31.8%)	85/281 (30.2%)	83/297 (27.9%)	0.8497

The p-values are the results of the analysis of variance, Kruskal–Wallis test, or chi-square test.

*AIS* Athens Insomnia Scale, *CES-D* Center for Epidemiologic Studies Depression Scale, *GEJE* 2011 Great East Japan Earthquake, *IES-R* Impact of Event Scale-Revised, *K6* Kessler Psychological Distress Scale, *LSNS-6* Lubben Social Network Scale.

*Mean ± standard deviation.

^†^Median and interquartile range (25–75 percentile).

^‡^Significantly higher or lower in the post-hoc test.

### Self-reported psychosocial indicators

**Figure 2:**
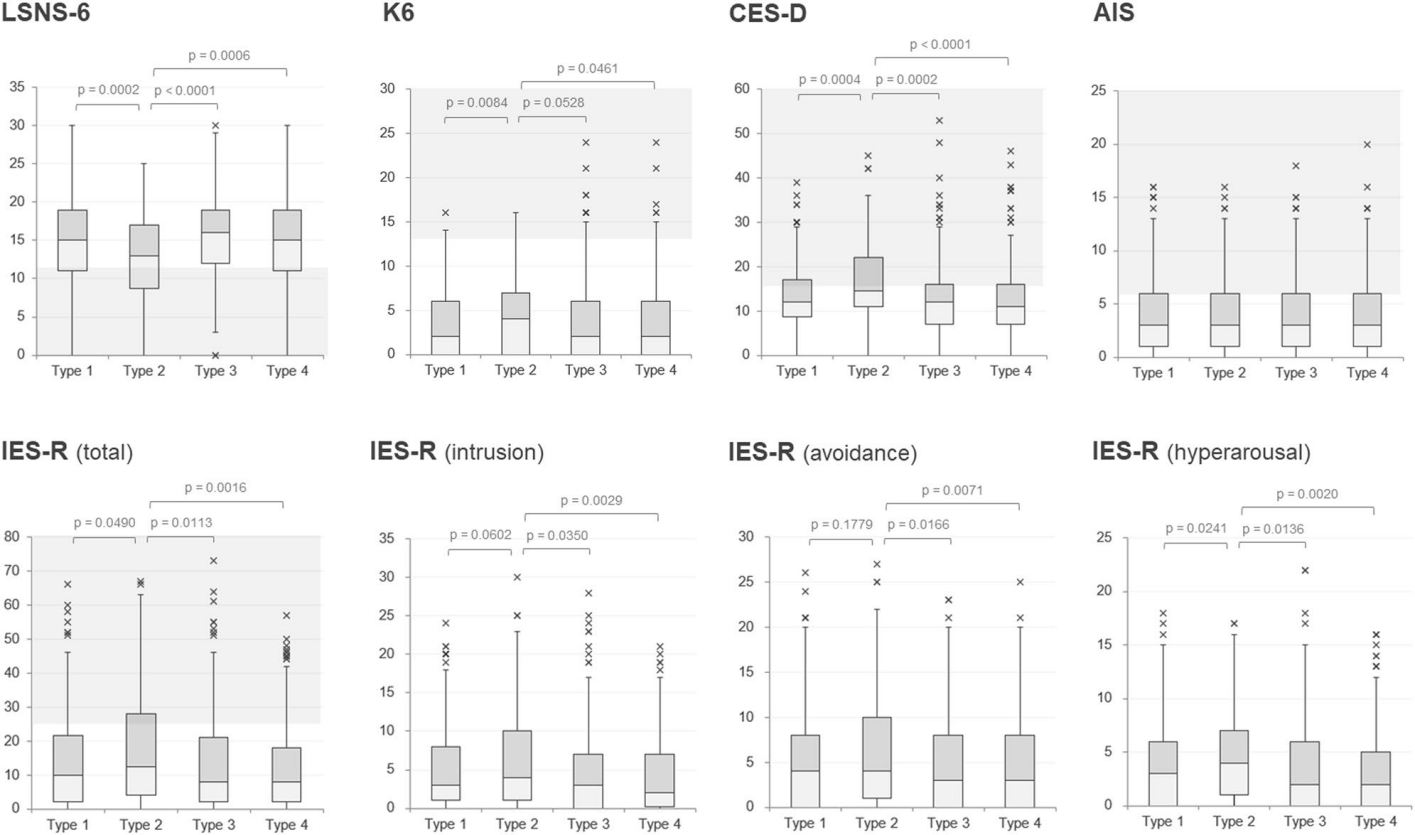
Boxplots for the scores of mental health-related measures by the current residential types. The four types of reconstructed residences are as follows: type 1, reconstruction on the same site (i.e., did not move); type 2, disaster-recovery public condominium; type 3, mass-translocation to higher ground with neighbors; and type 4, privately moving to remote areas. The boxes show the interquartile range (IQR, 25–75 percentile range), and bars represent $1.5\times IQR$. The plots outside the bars represent outliers. The p-values are the results of the Tukey–Kramer post-hoc test after the analysis of variance. The area filled with gray in each panel represents the scores suggesting abnormal psychosocial or mental health conditions. *AIS* Athens Insomnia Scale, *CES-D* Center for Epidemiologic Studies Depression Scale, *IES-R* Impact of Event Scale-Revised, *K6* Kessler Psychological Distress Scale, *LSNS-6* Lubben Social Network Scale.

**Figure 3:**
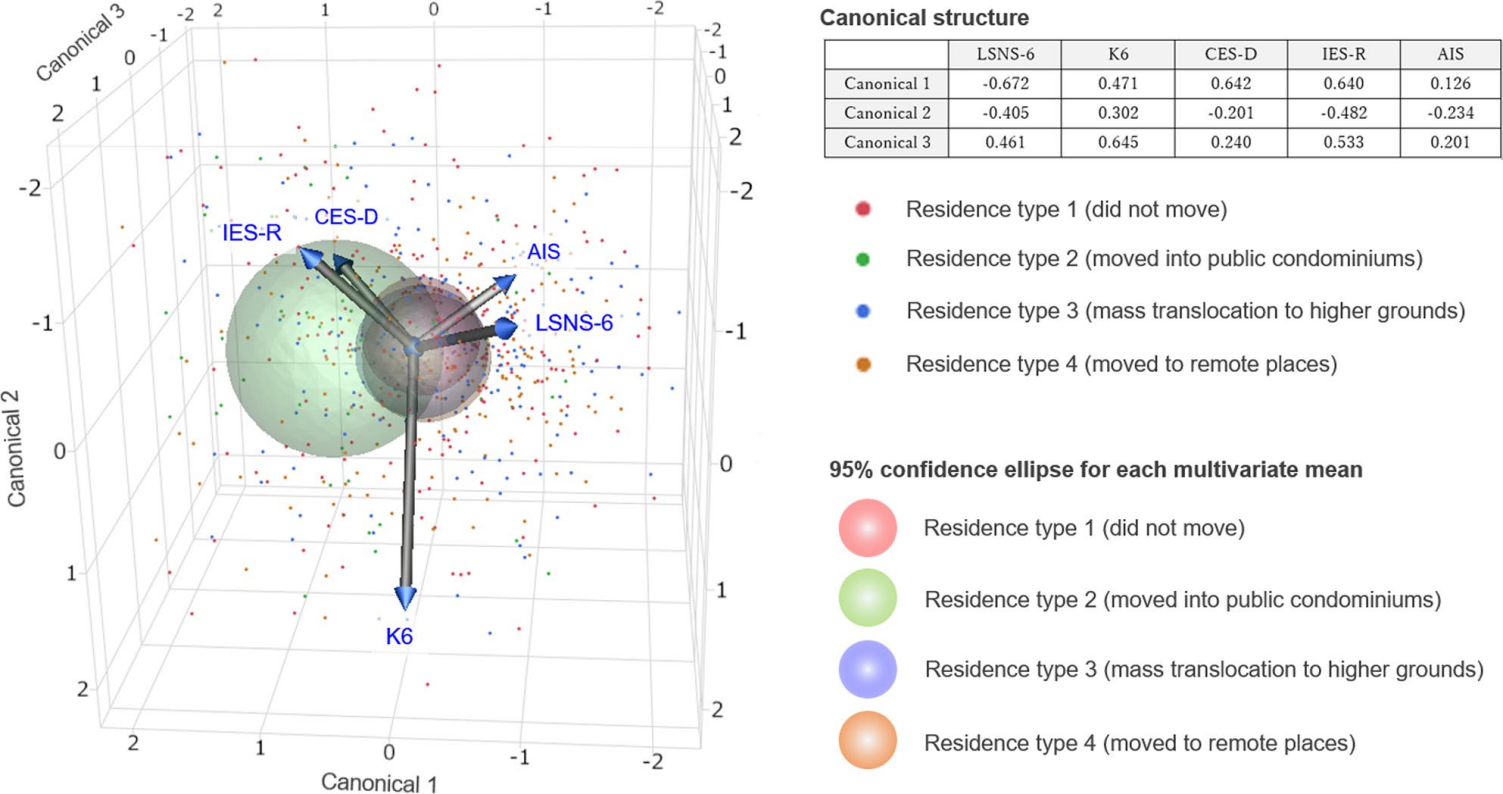
Canonical plot for a discriminant analysis of the psychosocial indicators by the current type of residences. Discriminant analysis was performed using the scores of the five self-reported psychosocial indicators as covariates and current type of reconstructed residences as the categorical variable. In the canonical plot, individual data are plotted, with the 95% confidence ellipse for the multivariate mean in each of the four types of reconstructed residences. The variable vectors show the correlations of the response variables with the three dimensions of the canonical space. *AIS* Athens Insomnia Scale, *CES-D* Center for Epidemiologic Studies Depression Scale, *IES-R* Impact of Event Scale-Revised, *K6* Kessler Psychological Distress Scale, *LSNS-6* Lubben Social Network Scale.

To evaluate the current psychosocial and psychiatric conditions of the displaced population, valid scores without missing data for each sub-item of the evaluated psychosocial indicators were obtained from 1048 (97.9%) of the eligible participants for the LSNS-6, 1032 (96.4%) for the K6, 799 (74.6%) for the CES-D, 971 (90.7%) for the IES-R, and 1041 (97.2%) for the AIS. General social interaction activities, estimated from the scores of the LSNS-6, were significantly lower (i.e., worse) among those who moved into disaster-recovery public condominiums (p < 0.0001, analysis of variance [ANOVA]). Of the evaluated psychiatric indicators, the scores of the CES-D (p < 0.0001, ANOVA) were significantly higher (i.e., worse) among those who moved into disaster-recovery public condominiums. The scores of K6 (p = 0.0345, Kruskal–Wallis test) and IES-R (p = 0.0281, Kruskal–Wallis test) were slightly higher (i.e., worse) in the public condominium group; however, the difference was not statistically significant (p < 0.01) in this study. Meanwhile, the AIS scores (p = 0.7572, ANOVA) did not differ according to the type of reconstructed residence. Box and whisker plots for these measures by the types of residence are depicted in Fig. 2. To graphically represent the different profiles of the psychosocial indicator scores according to the types of current residences, a three-dimensional canonical plot derived from discriminant analysis that employed scores of the five self-reported psychosocial indicators as covariates and current type of reconstructed residences as the categorical variable is depicted in Fig. 3. The obtained canonical plot showed a separation of the 95% confidence ellipse for public condominium group from ellipses for each of the other three types of current residences.

Next, to adjust for the possible confounding effects of the current socio-economic factors on the self-reported psychosocial indicators, an analysis of covariance (ANCOVA), by employing the previous history of living in post-disaster prefabricated temporary housings, the current employment status, and the number of family members per household as covariates, was performed for each psychosocial indicator. The resulting scores of the LSNS-6 (p < 0.0001) and CES-D (p < 0.0001) remained significantly worse in the public condominium group. The scores of K6 (p = 0.0941) and IES-R (p = 0.0244) were slightly higher (i.e., worse) in the public condominium group; however, the difference was not statistically significant. The AIS scores (p = 0.5677) did not differ between the four residential types.

### Correlation matrix within the five psychosocial indicators

To investigate the backgrounds behind the absence of difference in the AIS scores between the four types of reconstructed residence, correlation matrices within the five self-reported psychosocial indicators were built for the whole study participants and for those living in disaster-recovery public condominiums. The matrices are shown in Table 2. For both populations, the scores of the LSNS-6 showed no or only weak correlations with the other self-reported indicators. The overall patterns of the correlations among the five indicators were largely the same between the whole study participants and those living in public condominiums.

**Table 2:** Correlation coefficients between the five self-reported psychosocial indicators.

	LSNS-6 (n = 1048)	K6 (n = 1032)	CES-D (n = 799)	IES-R (n = 971)	AIS (n = 1041)
**In the whole study participants (n = 1071)**					
LSNS-6	–	*ρ* = − 0.209	*ρ* = − 0.297	*ρ* = − 0.053	*ρ* = − 0.187
K6	(p < 0.0001)	–	*ρ* = + 0.637	*ρ* = + 0.581	*ρ* = + 0.584
CES-D	(p < 0.0001)	(p < 0.0001)	–	*ρ* = + 0.597	*ρ* = + 0.542
IES-R	(p = 0.1039)	(p < 0.0001)	(p < 0.0001)	–	*ρ* = + 0.454
AIS	(p < 0.0001)	(p < 0.0001)	(p < 0.0001)	(p < 0.0001)	–
**In the public condominium group (n = 118)**					
LSNS-6	–	*ρ* = − 0.260	*ρ* = − 0.185	*ρ* = − 0.101	*ρ* = + 0.024
K6	(p = 0.0066)	–	*ρ* = + 0.720	*ρ* = + 0.614	*ρ* = + 0.607
CES-D	(p = 0.0989)	(p < 0.0001)	–	*ρ* = + 0.640	*ρ* = + 0.484
IES-R	(p = 0.3067)	(p < 0.0001)	(p < 0.0001)	–	*ρ* = + 0.427
AIS	(p = 0.0239)	(p < 0.0001)	(p < 0.0001)	(p < 0.0001)	–

The shown values are the Spearman’s correlation coefficients (*ρ*) between each of the evaluated mental health-related measures and the potential confounding factors. The p-values are the results of the test of no correlation. Missing data were handled by the pairwise deletion method.

*AIS* Athens Insomnia Scale, *CES-D* Center for Epidemiologic Studies Depression Scale, *IES-R* Impact of Event Scale-Revised, *K6* Kessler Psychological Distress Scale, *LSNS-6* Lubben Social Network Scale.

### Demographics and scores of psychosocial indicators

To evaluate the correlation between each of the evaluated background variables (i.e., sex, age, number of households, employment status, change in income) and the obtained scores of the self-reported psychosocial indicators, Spearman’s correlation coefficients (ρ) between the background data and the scores of the five self-reported psychosocial indicators were evaluated. The correlation matrix is shown in Table 3. None of the evaluated background factors were significantly correlated with the evaluated psychosocial or psychiatric measures.

**Table 3:** Correlation coefficients between the measured batteries and possible confounding background factors.

	LSNS-6 (n = 1048)	K6 (n = 1032)	CES-D (n = 799)	IES-R (n = 971)	AIS (n = 1041)
Sex (Male)	*ρ* = − 0.068 (p = 0.0276)	*ρ* = − 0.167 (p < 0.0001)	*ρ* = − 0.067 (p = 0.0569)	*ρ* = − 0.154 (p < 0.0001)	*ρ* = − 0.156 (p < 0.0001)
Age	*ρ* = + 0.067 (p = 0.0299)	*ρ* = + 0.085 (p = 0.0064)	*ρ* = + 0.160 (p < 0.0001)	*ρ* = + 0.166 (p < 0.0001)	*ρ* = + 0.030 (p = 0.0300)
Number of households	*ρ* = + 0.056 (p = 0.0718)	*ρ* = − 0.077 (p = 0.0134)	*ρ* = − 0.029 (p = 0.4146)	*ρ* = − 0.101 (p = 0.0016)	*ρ* = − 0.021 (p = 0.4991)
Employment	*ρ* = − 0.009 (p = 0.7619)	*ρ* = − 0.127 (p < 0.0001)	*ρ* = − 0.156 (p < 0.0001)	*ρ* = − 0.143 (p < 0.0001)	*ρ* = − 0.060 (p = 0.0525)
Decreased income after GEJE	*ρ* = − 0.003 (p = 0.9345)	*ρ* = + 0.061 (p = 0.1355)	*ρ* = + 0.096 (p = 0.0368)	*ρ* = + 0.152 (p = 0.0003)	*ρ* = + 0.107 (p = 0.0079)
History of living in prefabricated temporary housing	*ρ* = + 0.070 (p = 0.0359)	*ρ* = − 0.028 (p = 0.4047)	*ρ* = + 0.012 (p = 0.7566)	*ρ* = − 0.013 (p = 0.7079)	*ρ* = − 0.065 (p = 0.0544)

The shown values are the Spearman’s correlation coefficients (*ρ*) between each of the evaluated mental health-related measures and the potential confounding factors in the whole study participants. Missing data were handled by the pairwise deletion method.

*AIS* Athens Insomnia Scale, *CES-D* Center for Epidemiologic Studies Depression Scale, *IES-R* Impact of Event Scale-Revised, *K6* Kessler Psychological Distress Scale, *LSNS-6* Lubben Social Network Scale.

### Socio-economic factors and scores of psychosocial indicators

To evaluate the impact of employment status on the outcomes of this study, the current psychosocial and psychiatric conditions were further evaluated between the four types of residences and the current employment status. The results showed that the associations between psychosocial disturbances and life in disaster-recovery public condominiums were remarkable among the participants who were currently employed, as shown in Table 4. Based on this finding, Spearman’s correlation coefficients between employment status (currentt employment, income change) and other background demographics (age, sex, and number of people in the household) were evaluated. The current employment status was found to be significantly correlated with males (ρ =  + 0.24, p < 0.0001) and age (ρ = − 0.55, p < 0.0001). The current employment rate in the total enrolled younger adults aged < 65 years was 80.2% (n = 477/595), whereas in older adults aged ≥ 65 years, it was 26.5% (n = 120/453). The aforementioned results imply that the negative psychosocial impact of living in disaster-recovery public condominiums may be more remarkable in the younger age group with a higher employment rate.

**Table 4:** Psychosocial and psychiatric measures by the current types of residences and employment status.

	Types of the current reconstructed residence				p-value
	Did not move	Public condominium	Mass-translocation to higher ground	Privately moving to remote areas	
LSNS-6*					
Presently employed	15.1 ± 5.7	12.2 ± 4.7^‡^	15.3 ± 5.4	15.3 ± 5.4	0.0034
Unemployed	15.3 ± 6.0	13.0 ± 5.9^‡^	16.0 ± 5.8	14.9 ± 5.6	0.0115
**K-6**^†^					
Presently employed	2 (0–5)	3.5 (1–7)^‡^	1 (0–5)	2 (0–5)	0.0244
Unemployed	3.5 (0–6)	4 (0–7)	4 (0–7)	3 (0–6)	0.6764
CES-D*					
Presently employed	12.0 ± 6.9	17.4 ± 10.3^‡^	11.2 ± 6.8	12.0 ± 7.9	0.0014
Unemployed	14.6 ± 7.5	16.7 ± 8.2	15.1 ± 9.4	12.9 ± 8.7	0.0675
**IES-R**^**†**^					
Presently employed	8 (2–18)	12 (4–24.5)^‡^	5 (1–15.5)	7 (2–15)	0.0201
Unemployed	13.5 (6–24)	13.5 (4–29)	13 (4–25)	8 (3–20)	0.1400
AIS*					
Presently employed	3.9 ± 3.4	4.3 ± 4.1	3.6 ± 3.0	3.8 ± 3.1	0.6142
Unemployed	4.6 ± 3.9	4.4 ± 3.9	4.5 ± 4.0	4.3 ± 3.7	0.9503

The p-values are the results of the analysis of variance (for LSNS-6, CES-D, and AIS) or Kruskal–Wallis test (for K6 and IES-R) according to the distribution pattern in each variable.

*AIS* Athens Insomnia Scale, *CES-D* Center for Epidemiologic Studies Depression Scale, *IES-R* Impact of Event Scale-Revised, *K6* Kessler Psychological Distress Scale, *LSNS-6* Lubben Social Network Scale.

*Mean ± standard deviation.

^†^Median and interquartile range (25–75 percentile).

^‡^Significantly higher or lower in the post-hoc test.

Based on these findings, comparisons of the scores for the evaluated psychosocial indicators between the four types of residences were performed after stratifying the cohort into younger adults (aged < 65 years at the time of the survey) and older adults (aged ≥ 65 years at the time of the survey). The results showed worse scores in participants living in the disaster-recovery public condominiums for LSNS-6, K6, CES-D, and IES-R, which were all more remarkable in younger adults aged < 65 years than in older adults aged ≥ 65 years, as shown in Table 5.

**Table 5:** Psychosocial indicators by types of residences and age with the cutoff age of 65 years.

	Types of the current reconstructed residence				p-value
	Did not move	Public condominium	Mass-translocation to higher ground	Privately moving to remote areas	
LSNS-6*					
< 65 years old	14.7 ± 5.6	11.5 ± 4.9^‡^	14.9 ± 5.2	14.5 ± 5.3	0.0008
≥ 65 years old	15.9 ± 6.0	13.6 ± 5.6^‡^	16.8 ± 6.0	15.8 ± 5.8	0.0091
**K-6**^**†**^					
< 65 years old	2 (0–5)	4 (1–8)^‡^	1 (0–5)	2 (0–6)	0.0353
≥ 65 years old	3 (0–6)	4 (0–7)	3 (0–7)	2 (0–6)	0.2755
CES-D*					
< 65 years old	12.3 ± 7.3	16.2 ± 9.9^‡^	11.2 ± 7.3	12.4 ± 8.6	0.0041
≥ 65 years old	14.2 ± 7.1	18.0 ± 8.1^‡^	15.7 ± 8.9	12.6 ± 7.8	0.0023
**IES-R**^**†**^					
< 65 years old	7 (2–18)	12 (6–28)^‡^	6 (1–17)	7 (2–16)	0.0018
≥ 65 years old	16 (6–24)	14 (2–25)	14 (3–24)	9.5 (3–21)	0.1183
AIS*					
< 65 years old	3.9 ± 3.5	4.3 ± 3.9	3.8 ± 3.1	3.9 ± 3.0	0.8614
≥ 65 years old	4.3 ± 3.7	4.3 ± 4.0	4.1 ± 4.1	4.0 ± 3.8	0.9185

The data of the five self-reported psychosocial indicators were compared between the four types of reconstructed residences, after being divided into younger adults aged < 65 years and older adults aged ≥ 65 years. The p-values are the results of the analysis of variance (LSNS-6, CES-D, AIS) or the Kruskal–Wallis test (K-6, IES-R).

*AIS* Athens Insomnia Scale, *CES-D* Center for Epidemiologic Studies Depression Scale, *IES-R* Impact of Event Scale-Revised, *K6* Kessler Psychological Distress Scale, *LSNS-6* Lubben Social Network Scale.

*Mean ± standard deviation.

^†^Median and interquartile range (25–75 percentile).

^‡^Significantly higher or lower in the post-hoc test.

## Discussion

In this cross-sectional study with self-reported questionnaires, including indicators for social interaction activities and mental health conditions, the association between the current type of reconstructed residence and concurrent scores of the indicators was evaluated. The results indicated that moving into disaster-recovery public condominiums was associated with lower levels of social interaction and elevated rate of depressive state. This tendency was observed regardless of the current employment status or the age group, with a cutoff age of 65 years; however, it was especially remarkable among the younger adult disaster-affected population aged < 65 years. As the social isolation is an established risk of several mental health problems such as depression, the achieved results were reasonable findings^19,20^.

The results regarding moving into disaster-recovery public condominiums being associated with lower communication levels and worse mental health conditions may be explained by several hypotheses. A conceivable theory is that the majority of the residents in the sea-side area affected by the tsunami used to live in the old community and were accustomed to having close life-long relationships with neighbors^21^, which were suddenly destroyed by the disaster. They were unexpectedly forced to separate and relocate several times after the GEJE^22^. These social separation and isolation may have been accelerated by the environment of the public condominium, with its higher levels of security and privacy. Generally, residents living in a house are easily identifiable as being at home or not, and neighbors are able to communicate more easily with the residents^22^. In contrast, people who move to public condominiums find it difficult to be identifiable as being at home, and neighbors may refrain from casually visiting the residents. While the structure of public condominiums may be beneficial for maintaining privacy and security, it may deprive occasions of the social interactions.

Another finding to be discussed was the absence of difference in the AIS scores between the four types of reconstructed residences. To investigate the backgrounds, the correlation matrices within the five self-reported psychosocial indicators were evaluated in the whole participants and those living in public condominiums; however, the obtained patterns of the correlations were largely the same. This finding suggests that the association between sleep disturbance and other mental health disturbances may not be changed by a life in a public condominium. A possible hypothesis to explain the aforementioned finding may be that the discriminatory value of the AIS scores could be weak to show the small difference between the current types of residences, if any, as the range of the scores of AIS is relatively narrow with a positively-skewed distribution. However, the predictive and discriminatory values of AIS with a cutoff score of 6 for the patients with insomnia has been well established^23^, and such systematic problems based on the diagnostic characteristics of the AIS seems to be less likely. Another possible theory may be that the people with worse psychosocial or psychiatric conditions might have failed to answer to the questionnaires of AIS with missing or incomplete sub-item scores. Another possibility would be that there was virtually no difference in the prevalence of insomnia between the four residential types. This possibility seems to be reasonable, as the causal relationship or directionality between social isolation and sleep disturbance has not yet been established^24^.

A limitation of this study was that the exact amount of income of the disaster-affected population was not analyzed. It is likely that the amount of income may have influenced the decision to either reconstruct houses or move into more affordable public condominiums. Certainly, the relationship between lower socio-economic status and poor mental health conditions is known^25^. In this study, the current employment status and changes in the amount of income did not correlate with the current scores of the mental health-related questionnaire; however, this factor warrants further investigation. Another limitation was that this study did not investigate the details of the stressful experience or the caused economic and human damages by the disaster in each participant, which could have also influenced the current psychosocial and psychiatric conditions among the population.

In conclusion, disaster-affected residents who moved into disaster-recovery public condominiums, especially younger adults aged < 65 years with a high employment rate, are more likely to be affected by the potential negative psychosocial impact of living in disaster-recovery public condominiums. Effective intervention by national and local governments may be helpful to facilitate social interactions and better physical and mental health among the disaster-affected population who have moved into disaster-recovery public condominiums.

## Methods

### Study design

This study was a questionnaire-based cross-sectional observational survey performed five years after the occurrence of the GEJE on March 11, 2011. Adult residents of Shichigahama Town aged ≥ 18 years at the time of the study, whose houses were destroyed by the tsunami, were initially recruited in November 2016. The level of damage to the residences was uniformly and objectively judged by local government officials based on the standard building damage evaluation criteria defined by the Japanese government. Paper-based questionnaires to investigate the current type of reconstructed residence, current social interaction activities, and current psychosocial and mental health conditions were mailed to the candidates. From the respondents, individuals with missing data regarding the type of reconstructed residence were excluded from the analysis by applying the listwise deletion method. Missing data from other respondents with valid data regarding their current type of residence were handled by applying the pairwise deletion method in each statistical analysis.

### Indicator for the present social interaction activities

The levels of social networking and social interaction activities were assessed using the LSNS-6 to evaluate general social networking, along with seven self-reported questionnaires that were originally designed to evaluate specific social interaction activities. The LSNS-6 was originally developed by Lubben et al. in 2003 to evaluate social network level^14,26^. The scale comprises six self-reported questions, three of which are about relationships with family or relatives, while the other three relate to relationships with unrelated friends or acquaintances in the local community. It is scored from 0 to 30; a higher score indicating better social relationships. Scores < 12 suggest social isolation^27,28^.

### Indicators for the current mental health conditions

The current mental health conditions of the participants five years after the disaster were evaluated using the following questionnaires.

#### K6

The K6 assesses the psychological stress response of participants in the last 30 days. It comprises six categorical self-reported questions^15,29^. The higher the score, the more likely the subjects are to suffer from mood and anxiety disorders, including depressed states. In the present study, the standard K6 cutoff score of 13 or higher was applied to estimate the presence of psychological distress^30,31^.

#### CES-D

The CES-D was developed by the National Institute of Mental Health as a simple screening tool for patients with depression in epidemiological surveys^16,32^. This scale comprises 20 self-reported questions about depression-related physical and mental conditions in the last week of the participant. It is scored from 0 to 60; a higher score suggesting a more severe depressive state, with a cutoff score of ≥ 16^33,34^.

#### IES-R

The original version of the IES was developed by Horowitz et al*.* in 1979^35^, and later revised (IES-R)^17^. The scale evaluates the severity of a post-traumatic stress reaction in the participant’s last week. It comprises 22 self-reported questions—eight questions for evaluating intrusion-based symptoms, eight questions about avoidance-based symptoms, and six questions about hyperarousal-based symptoms. It is scored from 0 to 88, a higher score suggesting a higher likelihood of experiencing a post-traumatic stress reaction and a cutoff score being ≥ 25^17,36^.

#### AIS

The AIS was developed by the World Health Organization as a simple and reliable assessment tool for insomnia^18,37^. The scale comprises eight self-reported questions. It is scored from 0 to 24; a higher score indicating more severe insomnia. Usually, subjects with scores ≥ 6 are suspected to be suffering from insomnia^23,38^.

### Background demographic data

In addition to the above self-reported psychosocial indicators, we collected the following demographic data regarding the social and familial backgrounds of the participants: age, sex, number of family members per household, and current employment status at the time of the investigation. Furthermore, information about whether the amount of income decreased after the disaster was also investigated.

### Statistical analysis

Comparisons of quantitative data with normal distributions between the four groups by the type of the current reconstructed residence were performed using the ANOVA, followed by the Tukey–Kramer post-hoc test. Comparisons of the self-reported psychosocial indicators between the different types of current residences were further performed using an ANCOVA by employing the potential covariates with p < 0.10 in the univariate analyses (i.e., the current employment status, number of family members per household) to adjust for the current socio-economic factors. Comparisons of quantitative variables with non-normal distributions were performed using the Kruskal–Wallis test, followed by the Scheffe post-hoc test. Qualitative data were compared using the chi-square test. Because multiple comparisons were simultaneously performed in this study, statistical significance was set at p < 0.01. Discriminant analysis was performed using the scores of the five self-reported psychosocial indicators as covariates and current type of reconstructed residences as the categorical variable, and a canonical plot of the psychosocial indicators by the current type of residences was depicted. Statistical analyses were conducted using SPSS Statistics Base 22 software (IBM, Armonk, NY, USA) and R Statistical Software (version 4.0.5; R Foundation, Vienna, Austria). Discriminant analysis was performed using JMP Pro 16.0 (SAS Institute Inc., NC, USA).

### Ethics approval, consent to participate

This study was conducted in accordance with the current version of the Declaration of Helsinki, as revised in 2013. All participants gave written informed consent. The study was approved by the ethics committee of Tohoku University Graduate School of Medicine (approval number: THK-2011482).

## Abbreviations

AIS: Athens Insomnia Scale
CES-D: The Center for Epidemiologic Studies Depression Scale
IES-R: Revised version of Impact of Event Scale
GEJE: 2011 Great East Japan Earthquake
K6: Kessler psychological distress scale
LSNS-6: Lubben Social Network Scale
NIMH: National Institute of Mental Health

## References

[1] Umberson D, Montez JK (2010). Social relationships and health: A flashpoint for health policy. J. Health Soc. Behav..

[2] Cacioppo JT, Cacioppo S (2014). Social relationships and health: The toxic effects of perceived social isolation. Soc. Personal. Psychol. Compass.

[3] Bromley E (2013). Experiencing community: Perspectives of individuals diagnosed as having serious mental illness. Psychiatr. Serv..

[4] Tough H, Siegrist J, Fekete C (2017). Social relationships, mental health and wellbeing in physical disability: A systematic review. BMC Public Health.

[5] Yoshida H (2016). Post-traumatic growth of children affected by the Great East Japan Earthquake and their attitudes to memorial services and media coverage. Psychiatry Clin. Neurosci..

[6] Evans IEM (2018). Social isolation, cognitive reserve, and cognition in healthy older people. PLoS ONE.

[7] Teo AR, Choi H, Valenstein M (2013). Social relationships and depression: Ten-year follow-up from a nationally representative study. PLoS ONE.

[8] Mendoza-Nunez VM, Gonzalez-Mantilla F, Correa-Munoz E, Retana-Ugalde R (2017). Relationship between social support networks and physical functioning in older community-dwelling Mexicans. Int. J. Environ. Res. Public Health.

[9] Holt-Lunstad J, Smith TB, Layton JB (2010). Social relationships and mortality risk: A meta-analytic review. PLoS Med..

[10] Kuroda Y (2017). Occurrence of depressive tendency and associated social factors among elderly persons forced by the Great East Japan Earthquake and nuclear disaster to live as long-term evacuees: A prospective cohort study. BMJ Open.

[11] Hashimoto S (2017). Influence of post-disaster evacuation on incidence of metabolic syndrome. J. Atheroscler. Thromb..

[12] Tsuchiya N (2017). Impact of social capital on psychological distress and interaction with house destruction and displacement after the Great East Japan Earthquake of 2011. Psychiatry Clin. Neurosci..

[13] Nakaya N (2016). Prospect of future housing and risk of psychological distress at 1 year after an earthquake disaster. Psychiatry Clin. Neurosci..

[14] Lubben J (2006). Performance of an abbreviated version of the Lubben Social Network Scale among three European community-dwelling older adult populations. Gerontologist.

[15] Kessler RC (2002). Short screening scales to monitor population prevalences and trends in non-specific psychological distress. Psychol. Med..

[16] Shima S, Shikano T, Kitamura T, Asai M (1985). New self-rating scale for depression. Seishin Igaku (Clinical Psychiatry).

[17] Asukai N (2002). Reliability and validity of the Japanese-language version of the impact of event scale-revised (IES-R-J): Four studies of different traumatic events. J. Nerv. Ment. Dis..

[18] Soldatos CR, Dikeos DG, Paparrigopoulos TJ (2000). Athens Insomnia Scale: Validation of an instrument based on ICD-10 criteria. J. Psychosom. Res..

[19] Loades ME (2020). Rapid systematic review: The impact of social isolation and loneliness on the mental health of children and adolescents in the context of COVID-19. J. Am. Acad. Child Adolesc. Psychiatry.

[20] Wang J, Mann F, Lloyd-Evans B, Ma R, Johnson S (2018). Associations between loneliness and perceived social support and outcomes of mental health problems: A systematic review. BMC Psychiatry.

[21] Committee., S.-C.-S. E. Shichigahama Town 2008. *Shichigahama-cho-shi.* (2008).

[22] Shichigahama, T. O. *Shichigahama Reconstruction Overview*. (2017).

[23] Soldatos CR, Dikeos DG, Paparrigopoulos TJ (2003). The diagnostic validity of the Athens Insomnia Scale. J. Psychosom. Res..

[24] Griffin SC, Williams AB, Ravyts SG, Mladen SN, Rybarczyk BD (2020). Loneliness and sleep: A systematic review and meta-analysis. Health. Psychol. Open.

[25] Freeman A (2016). The role of socio-economic status in depression: Results from the COURAGE (aging survey in Europe). BMC Public Health.

[26] Kurimoto A (2011). Reliability and validity of the Japanese version of the abbreviated Lubben Social Network Scale. Nihon Ronen Igakkai Zasshi.

[27] Chang Q, Sha F, Chan CH, Yip PSF (2018). Validation of an abbreviated version of the Lubben Social Network Scale ("LSNS-6") and its associations with suicidality among older adults in China. PLoS ONE.

[28] Röhr S (2020). Changes in social network size are associated with cognitive changes in the oldest-old. Front. Psychiatry.

[29] Furukawa TA (2008). The performance of the Japanese version of the K6 and K10 in the World Mental Health Survey Japan. Int. J. Methods Psychiatr. Res..

[30] Prochaska JJ, Sung HY, Max W, Shi Y, Ong M (2012). Validity study of the K6 scale as a measure of moderate mental distress based on mental health treatment need and utilization. Int. J. Methods Psychiatr. Res..

[31] Kim G, DeCoster J, Bryant AN, Ford KL (2016). Measurement equivalence of the K6 scale: The effects of race/ethnicity and language. Assessment.

[32] Lewinsohn PM, Seeley JR, Roberts RE, Allen NB (1997). Center for Epidemiologic Studies Depression Scale (CES-D) as a screening instrument for depression among community-residing older adults. Psychol. Aging.

[33] Levine SZ (2013). Evaluating the seven-item Center for Epidemiologic Studies depression scale short-form: A longitudinal U.S. community study. Soc. Psychiatry Psychiatr. Epidemiol..

[34] Andresen EM, Malmgren JA, Carter WB, Patrick DL (1994). Screening for depression in well older adults: Evaluation of a short form of the CES-D (Center for Epidemiologic Studies Depression Scale). Am. J. Prev. Med..

[35] Horowitz M, Wilner N, Alvarez W (1979). Impact of Event Scale: A measure of subjective stress. Psychosom. Med..

[36] Lee SM, Kang WS, Cho AR, Kim T, Park JK (2018). Psychological impact of the 2015 MERS outbreak on hospital workers and quarantined hemodialysis patients. Compr. Psychiatry.

[37] Utsugi M (2005). Relationships of occupational stress to insomnia and short sleep in Japanese workers. Sleep.

[38] Okajima I, Nakajima S, Kobayashi M, Inoue Y (2013). Development and validation of the Japanese version of the Athens Insomnia Scale. Psychiatry Clin. Neurosci..

